# A comparative study on the micro-surface characteristics at black shale initial oxidation stage

**DOI:** 10.1038/s41598-020-67268-z

**Published:** 2020-06-26

**Authors:** Qi Li, Baolong Zhu, Jing Li

**Affiliations:** School of Civil Engineering and Architecture, Southwest University of Science and Technology, Mianyang, 621010 China

**Keywords:** Geochemistry, Element cycles

## Abstract

The pyrite oxidation is crucial to the overall black shale oxidation process. *A. ferrooxidans* was documented an effective oxidation ability on pure pyrite, but its role in black shale oxidation is unclear. In this study, a comparative study of acid solution and *A. ferrooxidans* on the micro-surface characteristics at the initial stage (7 days) was conducted on black shale slices, a comprehensive approach combining the micro-morphologies, micro-structures, micro-environmental pH and micro-surface elemental content were investigated by using polarizing microscopies, SEM, fluorescent staining and EDX line scan analysis. The pyrite oxidation rate was employed to the index for black shale oxidation degree, and analyzed by XRD, aqueous pH, oxidation-reduction potential (ORP), ferrous and ferric ions concentrations measurement. The results show that the micro-surface characteristics are different in acid solution and *A. ferrooxidans* groups, which significantly impact the pyrite oxidation rate. *A. ferrooxidans* promote the jarosite formation and elemental C accumulation on the rocks micro-surface, which is assumed to inhibit further reactions. Two reaction phases named “pyrite oxidized phase” and “jarosite formation phase” are proposed to occur in the initial stage of *A. ferrooxidans* oxidizing black shale. These findings provide experimental data to evaluate the micro-surface reactions during black shale oxidation process.

## Introduction

Black shale oxidation not only is geological significant in altering the Earth’s surface, but also is a critical stage in geochemical elemental recycling^1–8^. The sulfide minerals oxidation, primarily pyrite, is a key reaction during black shale oxidation process^9–13^. Jin *et al*.^11^ found that pyrite oxidation was the initial reaction occurred in the black shale oxidation process, which led to a great development in rock porosity. Phan *et al*.^12^ concluded that pyrite oxidation in black shale probably resulted from rock-water reaction, and accompanied by acid water production. Liao^14^ suggested that pyrite oxidation in black shale would promote other minerals dissolution because of acid erosion. Therefore, the pyrite oxidation rate is one of the most important index for black shale oxidation.

The pyrite is unstable when exposed to oxidants in the air or aqueous environment, Eqs. (1–3) illustrate the overall reactions of pyrite with oxidants of dissolved oxygen (DO) and ferric iron (Fe^3+^)^15^,

1 $${\text{FeS}}_{2}+3.5{\text{O}}_{2}+{\text{H}}_{2}\text{O}\to {\text{Fe}}^{2+}+2{{\text{SO}}_{4}}^{2-}+2{\text{H}}^{+}$$

2 $$4{\text{Fe}}^{2+}+{\text{O}}_{2}+4{\text{H}}^{+}\to 4{\text{Fe}}^{3+}+2{\text{H}}_{2}\text{O}$$

3 $${\text{FeS}}_{2}+14{\text{Fe}}^{3+}+{\text{H}}_{2}\text{O}\to 15{\text{Fe}}^{2+}+2{{\text{SO}}_{4}}^{2-}+16{\text{H}}^{+}$$

The pyrite is first oxidized by molecular oxygen to generate ferrous iron (Fe^2+^) (Eq. 1), then the Fe^2+^ ions convert to Fe^3+^ ions (Eq. 2), a more effective oxidant can accelerate the pyrite oxidation and hydrogen produced (Eq. 3)^16–18^. Given the Fe^3+^ is more stable in acid environment, the aqueous pH is recognized as an independent factor of pyrite oxidation^19^. On the other hand, experimental studies reveal that the intrinsic rate of abiotic conversion of Fe^2+^ to Fe^3+^ is very slow, but which can be promoted by some bacterial catalysis^20,21^. For example, *A. ferrooxidans*, an autotrophic bacterium which is abundant in pyrite ore bodies and their acidified drainages, can oxidize both ferrous iron and sulfur, is one of the most important microorganisms in pyrite oxidation^21–24^. Therefore, a more effective pyrite oxidation ability was reported by *A. ferrooxidans* than by acid solution^22^. However, most of these conclusions were based on experimental data from pure pyrite and only a long-term (more than 30 days) effect was considered^21,22,24–27^, these experimental interpretations can not be simply employed to the mechanistic comparison of chemical and biological oxidizing black shale, especially at the initial stage. In addition, black shale is low in porosity and permeability, the oxidation process is majorly determined by the rock surface reactions^12,28,29^. But most of previous studies were in regard to the minerals alternations and the macro-structure deformations^13,30–34^, the relationship between the micro-surface characteristics and the oxidation reaction was unclear.

Therefore, our interest here is in the correlation between micro-surface characteristics and the oxidation degree of black shale under chemical and biological effects at the initial stage. A comparative study of the effect of acid solution and *A. ferrooxidans* on the micro-surface characteristics after 7 days was conducted on black shale slices, a comprehensive approach combining the micro-surface characteristics including the micro-morphologies, micro-structures, micro-environmental pH and micro-surface elemental content were investigated. The pyrite oxidation rate was employed to the major index for evaluating the black shale oxidation degree, was analyzed by XRD, aqueous pH, ORP, ferrous and ferric iron concentrations. The results show that the micro-surface characteristics of black shale sample are different in acid solution and *A. ferrooxidans* groups, and these differences significantly impact the pyrite oxidation rate. Two reaction phases named “pyrite oxidized phase” and “jarosite formation phase” are proposed to occurred in *A. ferrooxidans* oxidizing black shale at the initial stage. These findings not only present new insight into the biological oxidizing black shale mechanism but also provide experimental data to evaluate surface reactions during this process.

## Results

### Micro-surface characteristics

**Figure 1:**
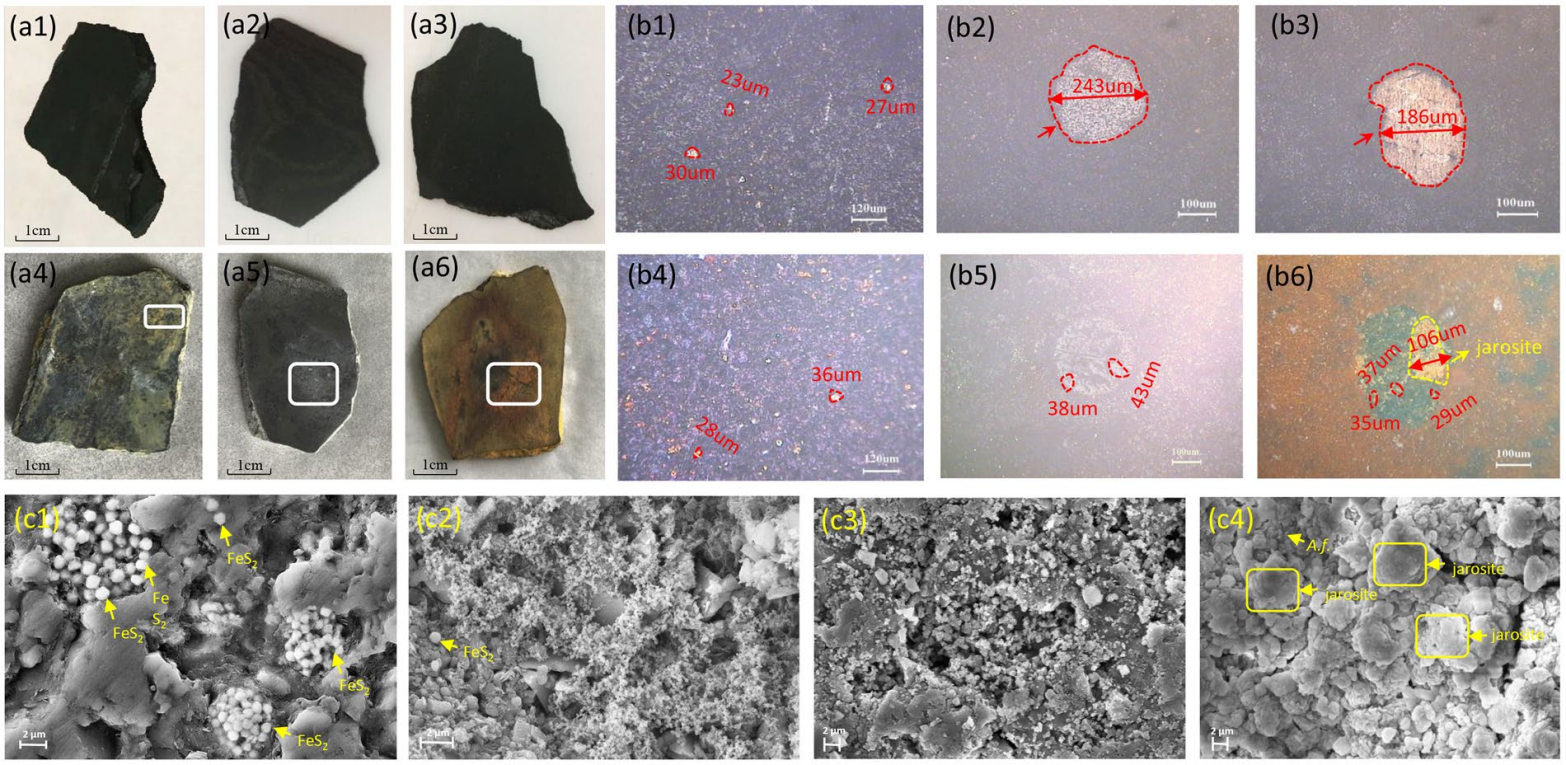
Black shale surface visual morphologies (**a1**–**a6**), polarizing microscope images (**b1**–**b6**) and SEM images (**c1**–**c4**). The (**a1**–**a3**), (**b1**–**b3**) and c4 are original state of samples, and the (**a4**–**a6**), (**b4**–**b6**) and (**c4**–**c6**) are samples after treatment in G1, G2 and G3. The white rectangles indicate the fade area in G1 (**a4**), the corrosion residual pit in G2 (**a5**) and the precipitate in G3 (**a6**) on the black shale surface. The red dashed circles in (**b1**–**b6**) indicate the pyrite assemblages and the yellow rectangles in (b6) indicate the jarosite precipitation on rock surface. The yellow rectangles in (**c4)** indicate the jarosite, and the yellow arrows in (**c1**,**c2**) indicate the pyrite grains, and yellow arrow in (**c4**) indicates the *A. ferrooxidans* cells. G1, Group 1, G2, Group 2, G3, Group 3, jarosite — KFe^3+^_3_(SO_4_)_2_(OH)_6_, *A.f* — *A.ferrooxidans* cells.

Figure 1 exhibits the rock morphologies obtained by visual, polarizing microscopes and SEM. The original samples are dark black and smooth, the spotted or assembled yellow-colored pyrite is heterogeneously distributed over rocks micro-surfaces. The major microstructures of black shale are coarse particle skeletons, a considerable amount of pyrite presents on the micro-surface as grains of a diameter of 0.3–0.5 µm. The rock morphologies significantly changed after treating with different aqueous systems for 7 days. In G1, a few fad areas are found on the rocks surface and the pyrite grains are fewer than that in the original state. The initial compacted and layered micro-structure becomes loosen because of the flocculent clay particles formation. In G2, the overall color of sample becomes shallow and several visible corrosive pits are observed on the rocks surface. The pyrite assemblages almost disappear after reactions, and an obvious circular eroded trace occurs on the rocks surface. A lot of irregular flocculent structures accompanied by large pores are present on the micro-surface. Whereas in G3, the rocks surface is covered with a large amount of tan and reddish-brown sediment after reactions. The pyrite assemblage is absent and left an obvious pit contained of yellow powdery deposits. There is a large amount of stacked compound with cauliflower-like shape covered on the rocks surface, which is identified as jarosite by EDX analysis. Liu *et al*.^35^ observed similar micro-morphologies on pyrite surface from K-jarosite biosynthesis system. Some short rod-like cells, probably *A. ferrooxidans*, are found at the rocks surface. These results also agree with the previous findings from Jiang *et al*.^22^. In addition, the medium in G1 and G2 is clear throughout the experiment, whereas in G3, the medium gradually turns to turbid from the 2^nd^ day, a lot of pale yellow and reddish brown precipitations are observed in the medium which is distinguished to be pure jarosite by XRD analysis.

**Figure 2:**
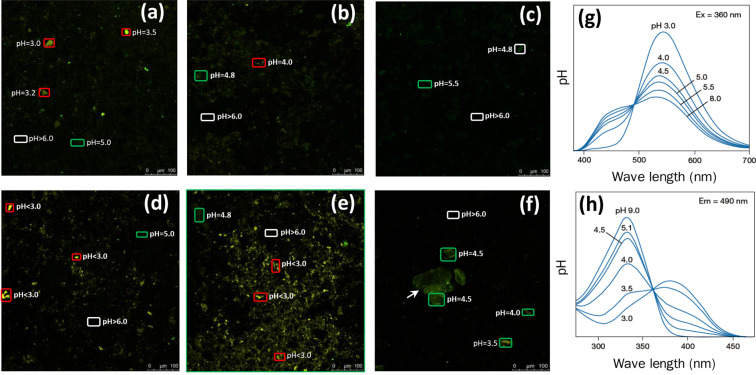
The MpH values by a pH-dependent fluorescent staining on the micro-surface of black shale samples before treatment in G1 (**a**), G2 (**b**) and G3 **(c**) and after treatment in G1 (**d**), G2 (**e**) and G3 (**f**). The calculated pH value of below 3.0, equal to 4.5 and above 6.0 were marked by red, green and white rectangles, respectively. The confocal microscopy observation was at emission wavelength at 360 nm (**g**) and excitation wavelength at 490 nm (h), respectively. MpH, micro-environmental pH values, G1, Group 1, G2, Group 2, G3, Group 3. The images are analysed by ImageJ software. ImageJ, Version: 2.0.0-rc-67/1.52c, URL: http://imagej.net/Contributors.

A pH dependent fluorescent dye, which excites yellow fluorescent color in acidic environment but turns to green when approached to neutral environment, is used for imaging the micro-environmental pH values (MpH) on the rocks surfaces. Due to the weathering reactions are initiated on the rocks surface, we assume that these reactions will change the micro-surface characteristics including the MpH. As shown in Fig. 2, the original micro-surface MpH is near neutral (pH > 6.0) although some low MpH regions (pH < 5.0) occurred (Fig. 2a–c). After treated by different aqueous systems for 7 days, the square of fluorescent density does not significantly change, but the low MpH areas increase compared with original images. In G1, the low MpH areas (pH < 3.0) increase by 10.8% and by 50.2% in G1 and G2, respectively (Fig. 2d,e). Whereas in G3, only the low MpH areas (pH = 4.5) increase by 9.2% (Fig. 2f) after reactions.

**Figure 3:**
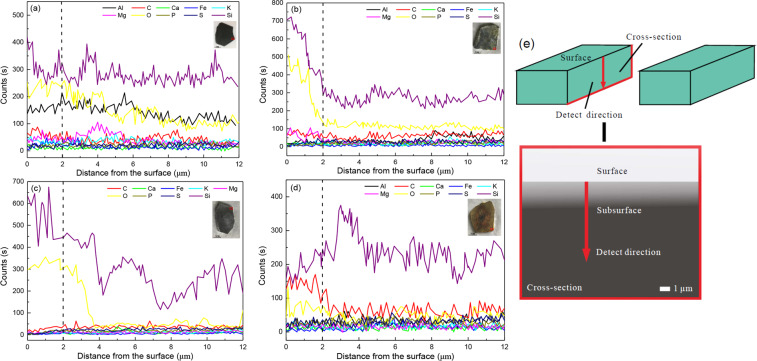
EDX line scans analysis of rock surface elements in the original state (**a**), and after treatment in G1 (**b**), G2 (**c**) and G3 (**d**), the dashed lines indicate the relative proportion of each element of in aspect of 2 µm depth. The schematic diagram (**e**) indicates the scan direction of the sample. G1, Group 1, G2, Group 2, G3, Group 3.

To probe the micro-surface elemental content, an EDX line scans analysis from the upper surface to the interior subsurface is conducted on the vertical plane of samples (Fig. 3e). In the original state, the elemental content at each point varies from the upper surface to the interior subsurface, among which the content of elemental Si, O and Al is relatively higher than other elements such as elemental C, S, Fe, Ca, K, and Mg (Fig. 3a). After treated by different aqueous systems for 7 days, the relative proportion of each element are different with respect to a 2 µm depth. The elemental Si and O remains a relatively high proportion, but for other elements, such as the elemental Al, S, Fe, Ca, K, and Mg, their proportion decreases than the original state (Fig. 3b). The G2 had the most decrease among groups, elements except for Si and O almost disappear with respect to a 2 µm depth (Fig. 3c). Whereas the relative proportion of elemental C are decreased in G1and G2, but increases in G3 (Fig. 3d). It is notably that, due to the special measure method, samples for original state measurement are not the experimental samples before treated, but obtained from the same parent rock. In spite of a little difference, it is reasonable to assume that this difference was same for three experimental groups and would not impact the conclusions.

### Mineral composition

The mineral composition of each sample is demonstrated in Table 1, the original samples mainly consist of quartz, plagioclase, muscovite, illite, calcite and pyrite. After treated by different solutions, the overall trend of minerals alteration is similar but the rate varies in three groups. The major minerals concentrations such as quartz, plagioclase, muscovite and calcite do not significantly change after reactions, but the pyrite content decreases by 15.1%, 48.1% and 64.2%, whereas the illite content increases by 26.9%, 41.6% and 32.4% in G1, G2 and G3, respectively. It can be seen that the jarosite only presents in G3 after treatment, which agrees with the SEM observations of numerals jarosite occurred on rocks micro-surface in G3.

**Table 1:** XRD analysis for minerals composition of black shale samples before and after treatment in each group. G1, Group 1, G3, Group 3. Q, quartz (SiO_2_).

Group	Q (%)	P (%)	C (%)	M (%)	I (%)	Cl (%)	P_y_ (%)	G (%)	J (%)	Co (%)
G1(before)	32.33	16.70	5.03	14.10	13.32	1.10	9.91	2.04	0.00	3.47
G1 (after)	32.04	16.49	4.54	14.16	16.91	1.16	8.42	1.89	0.00	3.39
G2(before)	34.43	17.30	3.03	17.40	15.32	1.93	7.08	2.12	0.00	1.39
G2 (after)	33.34	16.96	2.44	17.42	21.70	1.89	3.51	1.76	0.00	0.98
G3(before)	31.03	19.70	4.63	14.75	16.37	1.03	9.08	2.09	0.00	1.32
G3 (after)	29.83	18.16	3.81	14.11	21.68	1.05	3.25	1.73	7.86	0.52

Py, pyrite (FeS_2_). J, jarosite (KFe^3+^_3_(SO_4_)_2_(OH)_6_). P, plagioclase (NaAlSi_3_O_8_). I, illite (KAl_2_[(SiAl)_4_O_10_]·(OH)_2_·nH_2_O). C, calcite (CaCO_3_). Cl, clinochlore (Mg,Fe)_3_(Si,Al)_4_O_10_(OH)_2_·(Mg,Fe)_3_(OH)_6_. M, muscovite (KAl_2_ (AlSi_3_O_10_)(OH)_2_). G, goethite (FeOOH). Co, copiapite (Fe^2+^Fe^3+^_4_(SO_4_)_6_(OH)_2_·20H_2_O).

### Aqueous parameters

**Figure 4:**
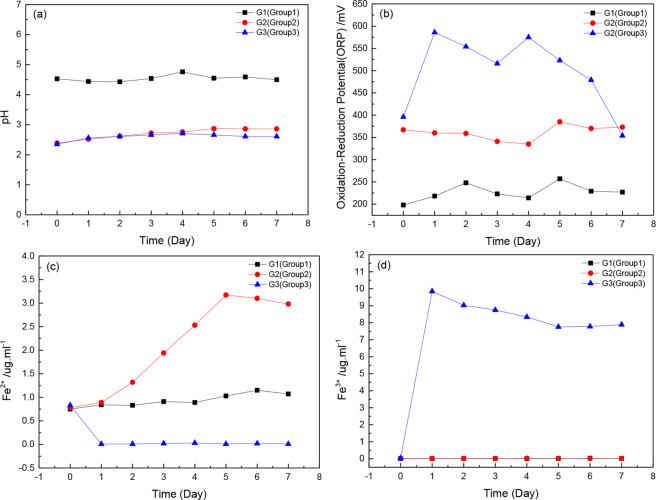
The aqueous pH (**a**), Oxidation-Reduction Potential (ORP) (**b**), Fe^2+^ ions (**c**) and Fe^3+^ ions (**d**) concentrations concentration during experiment.

The aqueous pH, oxidation-reduction potential (ORP), Fe^2+^ and Fe^3+^ ions concentrations are measured at every predetermined time interval, and the results are exhibited at Fig. 4. The aqueous pH values moderately change with reaction time, the initial values of pH are 4.5, 2.5 and 2.5 in G1, G2 and G3, respectively. Before the initial 4 days, the pH in three groups all slightly increase, whereas during the 5–7 days, the aqueous pH shows decreasing trend in G1 and G3 but increasing trend in G2, which probably because of the H^+^ consumption during the pyrite oxidation^35,35^(Fig. 4a).

The aqueous ORP reflects the rate of oxidation-reduction reactions, which is higher in G2 and G3 than in G1 at the initial time. Subsequently the ORP in three groups all increases, the G3 exhibits the highest increasing rate and reaches 586 at the 2^nd^ day. However, the ORP gradually decreases during the 3–7 days in G3, in spite of a re-bounce at the 4^th^ day. Whereas the ORP exhibits an increasing trend both in G1 and G2, and the values are much higher in G2 than that in G1 throughout the experiment (Fig. 4b).

The initial Fe^2+^ and Fe^3+^ ions concentrations are similar in three groups, the Fe^2+^ ions range from 0.75 mmol•L^−1^ to 0.83 mmol•L^−1^, and the Fe^3+^ ions are lower than 0.01 mmol•L^−1^. During the experiment time, the Fe^2+^ and Fe^3+^ ions concentrations show different trends among groups. The Fe^2+^ ions concentrations increase in G1 and G2, and reach to 1.15 mmol•L^−1^ and 3.17 mmol•L^−1^ at the 7^th^ day, respectively. However, the Fe^2+^ ions concentrations sharply decrease in G3 from the 2^nd^ day and keep to 0.01 mmol•L^−1^ throughout the experiment. In contrast, the Fe^3+^ ions concentrations are stable at 0.01 mmol•L^−1^ to 0.03 mmol•L^−1^ in G1 and G2 during the experiment, but significantly increases to 9.85 mmol•L^−1^ in G3 at the 2^nd^ day and keep above 7.76 mmol•L^−1^ throughout the experiment (Fig. 4c,d).

## Discussion

In natural systems, the black shale oxidation process is usually involved in multiple factors, such as chemical and biological mechanisms^29,36^. Previous studies have shown an effective pyrite oxidation ability presented in biological system such as *A. ferrooxidans*^22^, but few investigations have discussed its direct relationship to black shale oxidation. Considering the oxidation process initiated at the rock surface, we assumed a correlation between rock micro-surface characteristics alteration and oxidation degree, especially at the initial stage. Therefore, in this study, a comparative experiment is designed and carried out on black shale slices, the pyrite oxidation rate is employed to the index for black shale oxidation degree, aim to reveal a core difference in chemical and biological oxidizing black shale at the initial stage.

Based on the micro-surface morphologies result, it can be seen that both the acid solution and *A. ferrooxidans* are effective against pyrite oxidation. In G1 and G3, the pyrite assemblage almost disappeared after reactions, whereas it remains on the rocks surface in G1 (Fig. 1). The XRD results indicate that the most reduction of pyrite content is presented in G3. General speaking, the pyrite oxidation in black shale is accompanied by the microstructure decomposition associated with the damage of shale matrix cement^11,37^. However, this correlation seemed only occurred in abiolotic systems. The more robust pyrite oxidation led to the more clay minerals and irregular flocculent structures formed in G2 compared with that in G1. These results are consistent with previous findings^14^ that the acidic environment is facilitated to black shale oxidation. Whereas in the biolotic system, it is difficult to assess the micro-structure destruction, because of large amounts of jarosite covering on the rocks surface. Based on a field investigation, Liao *et al*.^14^ also reported a considerable amount of jarosite precipitation occurred on weathered black shale surface. Therefore, the relationship between micro-surface morphologies and the pyrite oxidation rate is different in abiolotic and biolotic systems.

Black shale is low in porosity and permeability, the oxidation process is controlled by “active” porosity which enables the fluid flow through into interior subsurface^11^. In the initial oxidation stage, we assume that the alterations of porosity and permeability would be limited to the micro-surface. However, the classic porosity measurement methods, such as nitrogen adsorption and high-pressure mercury intrusion tests seem unsuitable to the surface porosity measurement in this study. For example, previous studies by utilizing nitrogen absorption analysis documented the overall porosity distribution of black shale is major micro- and meso-pores with the pore diameter less than 50 nm, where fluid is difficult to flow through and led to further reaction^38^, but they are unable to distinguish the contribution of surface porosity, which may significantly different to the internal porosity. By using a novel fluorescent staining method, the fluid distribution on the rock surface can be illustrated, since this fluorescent dye can be detected only when it occurred in aqueous. We suppose that rinsing with neutral PBS can remove the fluid not contained in the rocks surface pores, therefore, the fluid distribution images can be employed to reveal the porosity on the rocks surface. Another feature of this dye is the color dependence on aqueous pH, which is yellow in acidic environment but turned to green when approach to neutral solution. Thus the MpH can also measure the pH of fluid distributed in the surface pores.

As demonstrated in Fig. 2, the fluorescent density is low in the original samples, indicating a low porosity of the rock surface. Although the total square of fluorescent density does not significantly change after reactions, the MpH is different to the original images and varies among groups. The low MpH areas increase more in G2 than that in G1, indicating more acid solution contained in the rocks surface pores in G2. These results agree with the alterations of micro-surface morphologies, the more pyrite oxidation results in more increase of acid solution permeability. But this interpretation can not apply to G3, in regard of a similar aqueous pH and pyrite oxidation rate presents in G2 and G3. The MpH does not significantly change in G3 by comparing with the original state, it probably since the surface jarosite coverage blocks the acid solution permeability. In addition, the carbonate also exists in the samples, its buffer ability may effect the MpH values, but considering the same experimental condition in all samples, this influence could be similar in three groups. Nevertheless, the distinct MpH among groups suggests the alternation of micro-surface porosity and permeability is different in biolotic and abiolotic systems.

Laboratory studies showed that the acid erosion could promote original matter oxidation and field investigations exhibited the organic matters nearly removed from the regolith^2,39^, these results indicated that the organic matters were depleted during black shale weathering process. Although a biological mechanism for the oxidation of pyrite by *A. ferrooxidans* has been intensively investigated^21–27^, very little work on its roles in organic matters oxidation process. Through EDX line scans measurement, we analysis the relative proportion of elements on the rocks micro-surface with aspect to a 2μm depth, to evaluate the elemental alteration on the rock micro-surface. As shown in Fig. 3, the relative proportion of elemental C, Fe, Mg, Al, S and K does not significantly change in G1, but markedly decreases in G2 after reaction. Although the overall content of minerals shows a small variation in G1 and G2, a more obvious minerals dissolution may occur on the rocks micro-surface in G2 in accordance with a more significant micro-structure destruction. Whereas in G3, which has a similar aqueous pH values with G2, the elemental C concentration increases after reactions. It may due to the attachment of *A. ferrooxidans* cell bodies on the rocks micro-surface, as numerous cell bodies are observed on the rocks micro surface in G3 (Fig. 1). Likewise, with the humidity climate of Pennsylvania, Jin *et al*.^11^ observed an addition of carbonate profile in the Rose Hill that may cause by bio-turbulence. Despite the EDX analysis just show the relative elemental C alterations, it can not equal to the total organic carbon (TOC) quantity, but for the micro-surface elemental C concentration, it shows depletion pattern in abiolotic system but addition pattern in biolotic system.

Previous literature documented a more effective oxidation ability presented in *A. ferrooxidans* compared with acid water alone, however, most of these approaches were inferred from pure pyrite^22^. If it assumed that the biological oxidation of black shale is much higher or even same the magnitude of the chemical effect, the overall rate of the biological oxidation processes should be re-estimated. However, based on field observations, the biological effect is usually limit to the rock surface^29^. The underlying mechanisms may relate to the different micro-surface alternations in chemical and biological oxidizing black shale, especially at the initial stage. In the proposed reactions at the initial oxidation stage, the pyrite embedded in black shale exhibited differential oxidation behavior in chemical and biological systems. In this regard, only effects of the first 7days are investigated in this study. Despite previous studies demonstrated a more obvious difference occurred in longer term experiments^22^, but which is not the focus of present study.

The pyrite oxidation rate is utilized as an index for black shale oxidation, which is higher in biolotic system at the initial 4 days. As shown in Table 1 and Fig. 4, the pyrite content decreases and the aqeoues ORP increases the most in G3, accompanied with the highest Fe^3+^ ions concentrations, these results are consistent with findings from pure pyrite^22^. However, the ORP and Fe^3+^ ions concentrations decreases from the 5^th^ day, indicating a deceleration of pyrite oxidation rate. Based on the results of micro-surface charateristics, this decrease is primarily attributed to the jarosite formation and covering on the rock surface.

The jarosite formation is a common problem of *A. ferrooxidans* bio-leaching engineering applications^21^, but is seldom reported to black shale biological oxidation mechanism. It is clear from above results that the jarosite formation is *A. ferrooxidans* dependence, the underlying mechanism may associate with following reasons. Initially, *A. ferrooxidans* cell surfaces served as nuclei for crystal growth of jarosite, subsequent cell metabolism will increase the amino acids, such as glycine and proline, which can significantly impact the morphology, yield and crystallinity of jarosite^40,41^. With the pyrite oxidation process ongoing, the ferric iron and sulfur concentrations increase, resulting in acceleration of jarosite formation^35,40^. Finally, the formed jarosite prefers to deposit on the rocks surface rather than dissolve in the biolotic aqueous systems^40^. Electro kinetic investigations have proposed that the changes in surface charge, such as a function of pH and ferric iron was the basis of pyrite oxidation^21,35^, however, the surface jarosite coating might passivate against this reaction. Additionally, the accumulation of carbonates on the rocks surfaces could also make a contribution to inhibit further reactions.

**Figure 5:**
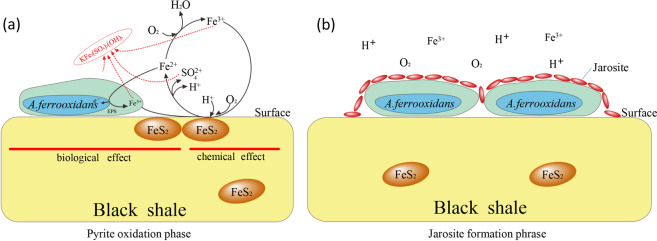
The schematic diagram of *A. ferrooxidans* biological weathering on black shale in pyrite oxidation phase (**a**) and jarosite formation phase (**b**). EPS, extracellular polymeric substances.

Therefore, two phases of reactions may involve in *A. ferrooxidans* oxidizing black shale at the initial stage. Firstly, *A. ferrooxidans* cells primarily attach on the rocks surface and then oxidize the ferrous iron into ferric iron, as well as the sulfur, in this phase, the pyrite oxidation rate is significantly prompted by *A. ferrooxidans* which is also recognized as “pyrite oxidized phase” (Fig. 5a). With the accumulation of ferric iron and sulfur, jarosite is formed and precipitated on the rocks surface, resulting in alteration of surface characteristics and inhibition of further reactions. Thus, this phase is so-called the “jarosite formation phase” (Fig. 5b)

It is notably that although a link between micro surface characteristics and black shale oxidation degree at the initial stage is founded in acid solution and *A. ferrooxidans*, it is still difficult to clearly interpret the difference in chemical and biological effects on black shale oxidation. For example, the pH in chemical system is at 2.50, which is much acid than bedrocks or regolith soils in natural black shale^13,14^. Furthermore, there are multiple sources of microorganisms presented in local environment except for *A. ferrooxidans*^42^, the contributions of other microorganisms should not be ignored which need further investigation. Finally, given there are multiple minerals contained in black shale, the future work will be focused on their roles in the overall oxidation process of black shale.

## Conclusion

In this study, we investigated the correlation between micro surface characteristics and the oxidation degree of black shale under chemical and biological effects at the initial oxidation stage. The oxidize rate of pyrite was employed to the major index for evaluating the black shale oxidation degree and a comparative study of acid solution and *A. ferrooxidans* was conducted on black shale slices. The results suggest,

(i) The microsurface characteristics vary in biolotic and abiolotic systems, the major cause for these differences is the jarosite formation and precipitation and the elemental C accumulation on the rocks microsurface, which is *A. ferrooxidans* dependence.

(ii) The pyrite oxidation in biolotic system undergoes an alternate of acceleration and deceleration at the initial oxidation stage, due to the alteration of microsurface characteristics.

(iii) Two reaction phases named “pyrite oxidized phase” and “jarosite formation phase” are proposed to *A. ferrooxidans* oxidizing black shale at the initial stage.

## Methods

### Samples preparation

The black shale samples were collected from a mountainous area in Dongan Town, Chengkou County, Chongqing City, China. The sample site was 10 m below the natural ground level, with the elevation about 1000 m above sea level and the latitude and longitude of 31°57’N and 108°37’E, respectively. The fresh rock fragments were cut into 2 cm (width) × 3 cm (length) × 0.3 cm (height) pieces, and carefully polished by a machine (MPJ-4, Xinyu, Shandong, China). Then all the samples were cleaned as previous described^14,21^, briefly, they were put into an ultrasonic cleaner with ethanol (75%, v/v) for 20 min followed by 5 times rinsing with deionized water, finally dried in a vacuum drying incubator at 40 °C and sterilized by UV irradiation for 12 h.

### Bacterial strain and culture media

*Acidithiobacillus ferrooxidans* (*A. ferrooxidans*) used in this study was isolated from the acidic groundwater near the sampling site, the strain was cultured in 9 K medium containing (per litre): 3 g (NH_4_)_2_SO_4_, 1 g KCl, 0.5 g K_2_HPO_4_, 0.5 g MgSO_4_·7H_2_O, 0.001 g Ca(NO_3_)_2_, and 44.2 g FeSO_4_·7H_2_O, the pH of medium was adjusted to 2.50 by using 0.1 M H_2_SO_4_. After being cultivated to the logarithmic growth phase, the suspensed *A. ferrooxidans* cells were filtered by a 3 um pore-size filter to remove the solid material in the medium, and then the filtrate was harvested by centrifugation (2376 × g, 30 min). The isolated cells were resuspended into a iron-free 9 K medium with pH at 2.50, and adjusted the cell density to 4 × 10^8^ cells/mL by counting with a haemocytometer.

### Experimental set-up

Three systems of bath groups were performed in this study, a 9 K medium without ferrous sulphate as (per litre): 3 g (NH_4_)_2_SO_4_, 1 g KCl, 0.5 g K_2_HPO_4_, 0.5 g MgSO_4_·7H_2_O and 0.001 g Ca(NO_3_)_2_ was used for all experiment groups. In two abiotic groups, the initial pH of the medium was different, where the Group 1 (G1) was 4.50 and the Group 2 (G2) was adjusted to 2.50 by using 0.1 M H_2_SO_4_. In biotic Group 3 (G3), the initial pH of medium was adjusted to 2.50 by using 0.1 M H_2_SO_4_ and inoculated with 10% (v/v) *A. ferrooxidans* cells. All samples were immersed in a 250-mL Erlenmeyer flask and incubated in a constant temperature shaker at 28 °C, 150 r/p.

### Microscopic analysis

The polarized light microscopy (Olympus, Tokyo, Japan) was used for rock surface mineral observation before and after treatment. Small dried fragments of samples before and after treatment were fixed onto aluminum stubs and sputter-coated with gold, then examined with a scanning electron microscopy (SEM) equipped with an energy dispersive X-ray spectroscopy (EDX) (Zeiss, Heidenheim, Germany). The working condition of SEM was at acceleration voltage of 20 kV, extraction voltage of 4.9 kV and emission current of 10 μA. The EDX was operated at an excitation voltage of 20 kV, and the processing time for dead time of 35% to 40%, the acquisition time was 100 s (point analysis) and 1800 s (line scan) with the energy range of 0 ~ 20 keV. However, due to the special requirements of sample size and preparation methods, it is hard to conduct on same sample before and after experiments for SEM and EDX analysis, therefore, samples obtained from the same parent rock were employed to the original state measurement.

### Rock surface micro-environmental pH (MpH) staining

A fluorescent pH indicator, LysoSensor Yellow/Blue Dye (Thermo-Fisher, San Jose, CA, USA), was utilized to measure the rock surface MpH by staining the black shale samples before and after treatment. The experiments were conducted following the manufacturer’s instructions, briefly, the samples were firstly slightly rinsed by PBS (pH 7.0) for three times, then incubated at the working solution (1 µmol) for 5 minutes. After rinsing by PBS (pH 7.0) once again, the MpH was observed by using a confocal fluorescence microscope (Leica, Solms, Germany) with emission and excitation wavelengths of 490 and 360 nm (Fig. 2g,h). The images were analysed by Image J software.

### XRD analysis

The XRD diffraction analysis (Bruker, Karisruhe, Germany) was performed using Cu Kα radiation (λ = 1.54060) at a voltage of 40 kV and a current of 40 mA, with a scanning range 2.5∼80° (2θ) and a scanning step length of 0.01° (2θ). A Cu filament and Ni filter was performed for the qualitative mineral analysis.

### Aqueous parameter analysis

Aqueous samples of 2 mL each were extracted at every 24 hours for pH (HACH, Loveland, CO, USA), dissolved oxygen (DO) (Mettler Toledo, Zurich, Switzerland) and oxidation-reduction potential (ORP) (HACH, Loveland, CO, USA) measurements. For the ferric and ferrous ions measurement, samples were firstly filtered by a cellulose acetate membrane (0.22um pore-size), the *o*-phenanthroline method was then utilized and performed on an ultraviolet visible spectrophotometer (Thermo-Fisher, San Jose, CA, USA) at λ = 510 nm^43^.

## Data Availability

All results and data of this article are available in the paper.
